# Fostering innovation and sustainable thinking in surgery: an evaluation of a surgical hackathon

**DOI:** 10.1308/rcsann.2024.0010

**Published:** 2024-04-02

**Authors:** Z Ahmed, A Zargaran, D Zargaran, J Davies, A Ponniah, P Butler, A Mosahebi

**Affiliations:** ^1^ University College London, UK; ^2^ Royal Free London NHS Foundation Trust, UK; ^3^ UCL Global Business School for Health, UK

**Keywords:** Artificial intelligence, Hackathon, Innovation, Machine learning, Plastic surgery, Sustainable surgery

## Abstract

**Introduction:**

Surgery represents a major source of carbon emissions, with numerous initiatives promoting more sustainable practices. Healthcare innovation and the development of a digitally capable workforce are fundamental in leveraging technologies to tackle challenges, including sustainability in surgery.

**Methods:**

A surgical hackathon was organised with three major themes: (1) how to make surgery greener, (2) the future of plastic surgery in 10 years, and (3) improving healthcare outcomes using machine learning. Lectures were given on sustainability and innovation using the problem, innovation, market size, strategy and team (PIMST) framework to support their presentations, as well as technological support to translate ideas into simulations or minimum viable products. Pre- and post-event questionnaires were circulated to participants.

**Results:**

Most attendees were medical students (65%), although doctors and engineers were also present. There was a significant increase in delegates' confidence in approaching innovation in surgery (+20%, *p* < 0.001). Reducing waste packaging (70%), promoting recyclable material usage (56%) and the social media dimension of public perceptions towards plastic surgery (40%) were reported as the most important issues arising from the hackathon. The top three prizes went to initiatives promoting an artificial intelligence-enhanced operative pathway, instrument sterilisation and an educational platform to teach students research and innovation skills.

**Conclusions:**

Surgical hackathons can result in significant improvements in confidence in approaching innovation, as well as raising awareness of important healthcare challenges. Future innovation events may build on this to continue to empower the future workforce to leverage technologies to tackle healthcare challenges such as sustainability.

## Introduction

Surgery and healthcare services worldwide account for a significant proportion of global carbon emissions. In the United Kingdom, the National Health Service (NHS) is responsible for 25% of public sector emissions.^1^ Waste is also a large environmental burden; hospitals and laboratories produce more than 5 million tonnes of waste per annum with a high dependency on plastic, 95% of which is not recovered.^2,3^ The COVID-19 pandemic further heightened waste, and subsequent analysis of unnecessary unrecyclable waste production highlighted the need for change.^4–6^ The UK government is targeting net carbon zero by 2050 and the United Nations Climate Change conference COP26 stated its aim to keep global warming to a maximum of 1.5°C. In 2022, the Intercollegiate Green Theatre Checklist was released as part of a joint initiative from the Royal Colleges in the UK. This highlights the urgent need for increased environmental sustainability in surgery and healthcare.^7,8^

Three main carbon hotspots in the operating theatre may be considered: anaesthetic gases, consumables and energy. These provide actionable waste reduction targets where efforts can be concentrated to increase the environmental sustainability of the operating room. Although these provide a foundation for the development of sustainable surgical initiatives, there remains a significant gap between current and desired standards, and as such more work needs to be done to promote innovation across all career stages. Innovation, process redesign and re-engineering are all crucial to enable progression in surgery and environmental sustainability.^9,10^ We hosted a hackathon to encourage innovation in surgery with environmental sustainability as one of the key themes.

A hackathon brings people together to engage and collaborate with the aim of finding solutions to a problem. The hackathon was held over two days in conjunction with the plastic surgery department at the Royal Free Hospital in London. Delegates included both medical and non-medical professionals who worked together to develop innovations that they pitched to judges with the prospect of receiving funding to fulfil their ideas.

The aim of this study is to assess the success of hackathons in promoting innovations in environmental sustainability in medicine and surgery, and to demonstrate how hackathons can encourage innovations to reduce the environmental burden of healthcare.

## Methods

This paper is based on a literature review and report of a hackathon designed to encourage innovations in plastic surgery.

### Hackathon set-up

The hackathon was held at the Royal Free Hospital in London in conjunction with the plastic surgery department over two days in November 2022. It was advertised to target stakeholders with an interest in medical innovation and to delegates from medical and non-medical backgrounds. Delegates were provided with the choice of three problems to solve:

• how to make surgery greener;
• the future of plastic surgery in 10 years;
• using machine learning, how can we improve healthcare outcomes in clinical practice.

Delegates were also provided with example problems and solutions for consideration. These included: reducing waste packaging; optimising dressing care regimens; delivering specialist tertiary care in the community; promoting recyclable material usage; promoting safety culture in non-surgical aesthetics; leveraging technologies in pressure ulcer management; enhancing the consent process in plastic surgery; and finally, the social media dimension in the public perception towards plastic surgery.

Over two days, 64 delegates networked, formed teams and came up with innovations. Delegates were provided with a problem, innovation, market size, strategy and team (PIMST) framework to help them design their innovation and pitches.

Teams then pitched their innovations to judges comprising senior consultants and professors of plastic surgery, investors and a professor from UCL's Global Business School for Health. Judges marked the pitches based on the originality of an idea, the primary research behind it, the impact of the problem, a multidisciplinary approach and the presentation itself. There was a first and a second prize, with an additional prize for the best innovation focusing on machine learning and artificial intelligence (AI). This prize was awarded as part of a new initiative called the Royal Free Accelerator for Intelligent Surgery and Enterprise (RAISE). The objectives of RAISE are to seek out talented young people with a passion for innovation in surgery and healthcare, and to support them by providing funding and mentorship to help them fulfil their healthcare business ventures.

### Hackathon follow-up

Following the hackathon, a questionnaire was circulated to assess which issues delegates felt were most important regarding innovation in surgery and healthcare, and which issues they already had ideas for prior to the hackathon. Delegates' confidence in approaching healthcare innovation and their perceptions of the importance of innovation in surgery and medicine before and after the hackathon were assessed. Delegates' experience using the PIMST framework and their perception on the need for collaboration with non-medical professions to drive surgical change were also evaluated. Answers were graded on a Likert scale of 1 (not important at all) to 5 (extremely important).

### Statistical analysis

The professional backgrounds of delegates were recorded as percentages. Questionnaire responses for delegates' perceptions of innovation, the need for collaboration and use of the PIMST framework were all found to be non-normally distributed (*p* < 0.001 and *p* = 0.02, respectively, in the Shapiro–Wilks test and Kolmogorov–Smirnov tests for normality). Therefore, the median result for each question was calculated and presented with quartiles. The frequency of each example innovation that delegates felt was most important and how many ideas delegates already had prior to the hackathon were recorded. Furthermore, the Wilcoxon signed-rank test was used to compare the median result for people's self-assessed confidence in approaching healthcare innovation before and after the event, with *p* < 0.05 being classed as significant.

## Results

### Delegates' backgrounds

Of the 64 delegates who attended the hackathon, 43 completed the post-hackathon questionnaire (67% delegates). Delegates who did not complete the questionnaire did not have significantly different backgrounds from those who did complete the questionnaire, as verified by an attendance register recorded during the hackathon.

Most delegates who attended the hackathon were medical students (65%; Table 1), and because of the location of the event, which was held in North London, came from different medical schools mainly concentrated in London. The majority of other delegates were either Foundation Year doctors or surgeons in training. Only 5% of delegates were from a non-medical background.

**Table 1:** Delegates' professional backgrounds

Background	*N* (%)
Total responses	43 (100)
Medical student	28 (65)
Foundation year doctor	6 (14)
Core trainee	3 (7)
Registrar	4 (9)
Engineer	2 (5)

### Delegates' questionnaire responses

The median delegates' response to the need for collaboration with non-medical professions to drive surgical change was rated 4 (important), which was notable given that almost all delegates at the hackathon were from a medical background (Appendix). The median score for how useful people found the PIMST framework was also 4 (useful).

From the list of example innovations, reducing waste packaging was rated as the most important topic; 70% of delegates rated this issue as important (Table 2). Over half of delegates (56%) thought that promoting recyclable material usage among surgeons was an important issue. This highlights delegates' sense of urgency to make surgery more sustainable. There was also a correlation between the proportion of people who thought an issue was important and the proportion who already had ideas pertaining to this issue.

**Table 2:** Delegates' views on which example innovations were most important and which they already had ideas for prior to the hackathon

	Which topic(s) do you think are most important? *N* (%)	Which topic(s) did you already have ideas for before the hackathon? *N* (%)
Reducing waste packaging	30 (70)	17 (40)
Optimising dressing changing regimens	9 (21)	6 (14)
Delivering local community care in a specialist tertiary centre	11 (26)	8 (19)
Promoting recyclable material usage among surgeons	24 (56)	12 (28)
Promoting safety culture in non-surgical aesthetics	16 (37)	11 (26)
Leveraging technologies in pressure ulcer management	11 (26)	5 (12)
Enhancing the consenting process in plastic surgery	11 (26)	5 (12)
The social media dimension in public perceptions of, and behaviours towards plastic surgery	17 (40)	9 (21)

**Figure 1:**
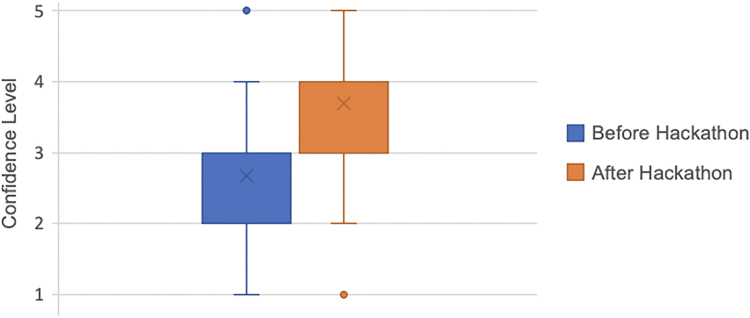
Delegates' confidence approaching innovation before and after the hackathon (1 = not confident at all, 5 = extremely confident)

The median value for delegates' confidence in approaching innovation before the hackathon was 3 (neither confident nor not confident). This increased by 20% to a median value of 4 (confident) after the two-day hackathon. The increase in confidence rating was significant when results were compared using the Wilcoxon signed-rank test (*p* < 0.001). When delegates were asked whether they felt that their discussions and innovations from the hackathon could impact the future of plastic surgery or surgery in general, the median response was 4 (likely) (Figure 1).

### Hackathon outputs

The first-place prize was awarded to a team that focused on AI in the operative pathway and second place was awarded to a team focusing on instrument sterilisation; third place was awarded for an educational platform to support research and innovation skills to promote sustainability.

## Discussion

This hackathon highlighted that making surgery and healthcare more sustainable is a key priority for current and future surgeons, doctors and healthcare providers’ agenda for change. This was demonstrated through delegates ranking “reducing waste packaging” and “promoting recyclable use among surgeons” most highly as issues they felt needed change. The hackathon was also shown to be a successful mechanism to empower participants with the skills and knowledge to be innovators. This is shown by the significant increase in delegates' confidence in approaching healthcare innovation following the hackathon. One explanation for the increased confidence may have been the PIMST model, which most delegates said they found useful during the hackathon. This highlights the value of providing strategies and frameworks to support and facilitate innovation.

Surgery is the most resource- and energy-intensive hospital sector and is a major contributor of waste.^11^ Strategies are being suggested for net-zero carbon surgery, but we are still a long way from achieving this.^12^ Hackathons, like the one described here, provide an opportunity to inspire new innovations towards net-zero carbon surgery. The group that received the second-place prize focused on redesigning instrument packs and personalising them to minimise the sterilisation of unused items. An audit performed at the University of California, Riverside found that an autoclave machine uses an average of 84kWh/day, which is extremely energy-intensive.^13^ Autoclaves also require significant volumes of water, 2,973L/day on average. Coupled with this, it is estimated that up to 80% of sterile instruments from an instrument pack opened in an operating theatre are not used.^14–16^ The members of the hackathon group addressed this problem by personalising the instrument packs based on procedure and surgeon preference using a web-based app. They performed a cost analysis and calculated that by reformulating the instrument packaging regimen, not only would there be a significant leap in surgical sustainability through a reduction of waste and energy, but also a huge financial benefit to the NHS and healthcare services worldwide.

Unlike many other industries, healthcare services have a 24-hour energy demand. With rising global energy costs, the NHS now faces an additional energy cost of £2 million per month.^17^ Adding this additional cost to an already stretched health service has potentially serious implications, and further urges the importance of generating new innovations to reduce energy costs for the NHS while simultaneously making energy sources greener.

To foster sustainability innovation, we must create a culture of innovation in surgery.^18^ We hypothesised that a hackathon would be a good starting point for this culture shift. A multicentre study of an extended 1–2-week hackathon for young healthcare innovators found a more than twofold increase in self-assessed innovation knowledge among participants (*p* < 0.001).^19^ One limitation of our hackathon was that it was only held over two days. However, the longevity of the hackathon is demonstrated from the majority of delegates stating that they thought it was likely that their discussions and innovations from the hackathon could impact the future of surgery. Furthermore, education is fundamental in empowering the current and future workforce to address such challenges, and to leverage available cutting-edge technologies to their maximum benefit. This was highlighted by one of the winning initiatives, which was an educational platform to help to address this, and future work could build on our foundation of the current hackathon to introduce and implement more educational resources to enable students and professionals to translate their ideas into viable products, incorporating established business theory.

To ensure that new healthcare innovations are sustainable, various frameworks have been proposed. Silva *et al* propose a framework encompassing a holistic approach to innovation including population health, health system, economic, organisational and environmental value.^20^ This represents a mutually beneficial innovation framework that could be incorporated as part of innovation education in medical curricula.

Interdisciplinary care between medical specialities is essential in healthcare. For surgical innovation, this collaboration must extend to non-medical professions. Another aim of our hackathon was to encourage collaboration between medical and non-medical professionals. This was reflected by the judges’ criteria for marking the pitches, which included points for a multidisciplinary approach. Previous healthcare hackathons have demonstrated the benefits that delegates gain in improving their collaboration skills with other professions.^21^ In our hackathon, although the median delegate score for the importance of collaboration driving surgical change was ranked as 4 (important), only 5% of delegates were from a non-medical profession. This is another limitation of our hackathon and may have been due to lack of advertising to these professions. In future, we would encourage further advertising to more professionals from computing, engineering and business backgrounds. Moreover, for future hackathons, we recommend refining criteria according to insights from the Centre for Sustainable Healthcare.

Many surgical and medical professional bodies have vouched for the importance of starting formal training in innovation. The Royal College of Surgeons of England has recently published an article detailing an Integrated Innovation Training Pathway that highlights early intervention points, whereby formal education in innovation could be integrated, ranging from undergraduate level to Post-Certificate of Completion of Training level.^22^ Furthermore, in the USA, the lack of education in healthcare innovation has led to the development of programmes such as the Surgery Innovation and Entrepreneurship Development Program in the University of Michigan^23^ and Surgical Innovation Committees.^24,25^ We propose that hackathons like ours are excellent starting points to ignite interest in surgical innovation across all training levels, but we also believe that further formal innovation training is needed, starting at a medical school level.

## Conclusion

In conclusion, our hackathon demonstrated that increasing surgical sustainability is a pressing issue for future surgeons, doctors and healthcare workers. The hackathon was also shown to be a good model to promote interest and provide resources to approach surgical innovation. Following on from our hackathon, more formalised training is needed on innovation in surgery to find sustainable solutions to current and future healthcare challenges and to enable continued evolution in healthcare.

## Acknowledgements

The hackathon was kindly sponsored by PLASTA, Amazon Web Services, Anatomyspots.com and PolyNovo.

## Data availability statement

Data are available upon reasonable request.

## References

[1] MacNeill AJ, Lillywhite R, Brown CJ. The impact of surgery on global climate: a carbon footprinting study of operating theatres in three health systems. *Lancet Planet Health* 2017; **1**: e381–e388. 29851650 10.1016/S2542-5196(17)30162-6

[2] Budd K. Hospitals race to save patients — and the planet. *AAMC News*. https://www.aamc.org/news/hospitals-race-save-patients-and-planet#:∼:text=The%20global%20health%20care%20industry,largest%20emitter%20of%20greenhouse%20gases (cited June 2024).

[3] Percival A. *Not so fantastic plastic*. NHS Providers. https://nhsproviders.org/news-blogs/blogs/not-so-fantastic-plastic (cited June 2024).

[4] World Health Organisation. *Tonnes of COVID-19 health care waste expose urgent need to improve waste management systems*. https://www.who.int/news/item/01-02-2022-tonnes-of-covid-19-health-care-waste-expose-urgent-need-to-improve-waste-management-systems (cited June 2024).

[5] World Health Organization. *Global analysis of health care waste in the context of COVID-19*. https://www.who.int/publications/i/item/9789240039612 (cited June 2024).

[6] Wise J. COVID-19: pandemic waste threatens human and environmental health, says WHO. *BMJ* 2022; **376**: o266. 35105547 10.1136/bmj.o266

[7] Department for Energy Security & Net Zero. *Review of net zero*. https://www.gov.uk/government/organisations/department-for-energy-security-and-net-zero (cited June 2024).

[8] GOV.UK. *COP26 explained*. https://www.gov.uk/government/topical-events/cop26#:∼:text=The%20COP26%20summit%20brought%20parties,UK%20Presidency%20for%20more%20information (cited June 2024).

[9] Kimble L, Rashad Massoud M. What do we mean by innovation in healthcare? *EMJ Innov* 2017; **1**: 89–91.

[10] Riskin DJ, Longaker MT, Gertner M, Krummel TM. Innovation in surgery. *Ann Surg* 2006; **244**: 686–693. 17060760 10.1097/01.sla.0000242706.91771.ce PMC1856601

[11] Rizan C, Steinbach I, Nicholson R *et al.* The carbon footprint of surgical operations. *Ann Surg* 2020; **272**: 986–995. 32516230 10.1097/SLA.0000000000003951

[12] Rizan C, Bhutta MF. Strategy for net-zero carbon surgery. *Br J Surg* 2021; **108**: 737–739. 33963828 10.1093/bjs/znab130

[13] Delphine Faugeroux Office of Sustainability, University of California Riverside. *Autoclave study*. https://www.priorclave.com/en-us/wp-content/uploads/sites/3/2018/08/LabDesign_Dec2016.pdf (cited June 2024).

[14] Nast K, Swords KA. Decreasing operating room costs via reduction of surgical instruments. *J Pediatr Urol* 2019; **15**: 153.e1–153.e6. 10.1016/j.jpurol.2019.01.013 30846251

[15] Stockert EW, Langerman A. Assessing the magnitude and costs of intraoperative inefficiencies attributable to surgical instrument trays. *J Am Coll Surg* 2014; **219**: 646–655. 25154669 10.1016/j.jamcollsurg.2014.06.019

[16] Farrokhi FR, Gunther M, Williams B, Blackmore CC. Application of lean methodology for improved quality and efficiency in operating room instrument availability. *J Healthc Qual* 2015; **37**: 277–286. 24112283 10.1111/jhq.12053

[17] Torjesen I. Exclusive: hospitals will be hit with “eye watering” energy bills this winter. *BMJ* 2022; **378**: o2088.

[18] Luis Felipe C, Laura V, Mauricio P, Lilian T. Re-discovering surgical innovation – An essential component of the academic surgeon. *Am J Surg* 2021; **222**: 905–908. 34016374 10.1016/j.amjsurg.2021.04.017

[19] Wang JK, Pamnani RD, Capasso R, Chang RT. An extended hackathon model for collaborative education in medical innovation. *J Med Syst* 2018; **42**: 239. 30328518 10.1007/s10916-018-1098-z

[20] Pacifico Silva H, Lehoux P, Miller FA, Denis J-L. Introducing responsible innovation in health: a policy-oriented framework. *Health Res Policy Syst* 2018; **16**: 90. 30200985 10.1186/s12961-018-0362-5 PMC6131953

[21] Pathanasethpong A, Areemit R, Teerakulpisut D *et al.* Health hackathon as a venue for interprofessional education: a qualitative interview study. *J Interprof Care* 2020; **34**: 832–834. 31865825 10.1080/13561820.2019.1696760

[22] Lam A, Bolton W, King M *et al.* The integrated innovation training pathway. *Bull R Coll Surg Engl* 2022; **105**: 12.

[23] Servoss J, Chang C, Olson D *et al.* The Surgery Innovation and Entrepreneurship Development Program (SIEDP): an experiential learning program for surgery faculty to ideate and implement innovations in health care. *J Surg Educ* 2018; **75**: 935–941. 28989009 10.1016/j.jsurg.2017.09.017 PMC5886837

[24] Biffl WL, Spain DA, Reitsma AM *et al.* Responsible development and application of surgical innovations: a position statement of the Society of University Surgeons. *J Am Coll Surg* 2008; **206**: 1204–1209. 18501819 10.1016/j.jamcollsurg.2008.02.011

[25] McNair LA, Biffl WL. Assessing awareness and implementation of a recommendation for surgical innovation committees. *Ann Surg* 2015; **262**: 941–948. 25373465 10.1097/SLA.0000000000001037

